# PyMine: a PyMOL plugin to integrate and visualize data for drug discovery

**DOI:** 10.1186/s13104-015-1483-3

**Published:** 2015-10-01

**Authors:** Rajan Chaudhari, Zhijun Li

**Affiliations:** Department of Chemistry & Biochemistry, University of the Sciences in Philadelphia, Philadelphia, PA 19104 USA

**Keywords:** PyMOL, Data integration, Data visualization, Drug discovery

## Abstract

**Background:**

Tremendous amount of chemical and biological data are being generated by various high-throughput biotechnologies that could facilitate modern drug discovery. However, lack of integration makes it very challenging for individual scientists to access and understand all the data related to a specific protein of interest.

**Findings:**

To overcome this challenge, we developed PyMine, a PyMOL plugin that retrieves chemical, structural, pathway and other related biological data of a receptor and small molecules from a variety of high-quality databases and presents them in a graphic and uniformed way.

**Conclusions:**

Developed as an interactive and user-friendly tool, PyMine can be used as a central data-hub for users to access and visualize multiple types of data and to generate new ideas intuitively for structure-based molecule design.

## Findings

### Background

Big data generated by a variety of high-throughput biotechnologies have catalyzed the booming of chemoinformatics and bioinformatics databases. The recent database issue of the *Nucleic Acid Research* journal has collected 171 such actively maintained databases [1] and many of them are growing at an enormous rate [2]. These databases cover various aspects of biological systems such as protein structures, functional sites, small-molecule ligands, and pathways etc., and thus are crucial for modern drug discovery. However, these data are stored in different databases and lack of data integration and visualization makes it challenging to effectively access and understand all the data in order to facilitate drug discovery processes [3]. This is especially true for a biology scientist not trained in bioinformatics who wish to adventure into the drug design avenue in their investigations as basic drug discovery is increasingly shifting toward academic laboratories [4].

Several attempts have been reported to address this challenge. Cytoscape is an open source software platform for complex network visualization and data integration [5]. UCSF Chimera is a molecular modeling program for visualization and analysis of molecular structures and related data [6]. Both Cytoscape and Chimera are stand-alone software packages and were not developed exclusively for drug discovery purpose. Aquaria [7] and PDBpaint [8] were developed to retrieve features from various databases and map them onto the protein structures. Both programs focus primarily on structural features such secondary structures and domains. NsSNP Loader (http://vbc.med.monash.edu.au/~fauxn/pymol_plugin/plugin.html) is a PyMOL plugin that maps the non-synonymous single nucleotide polymorphism (nsSNP) data on the structure of the protein. The PDBbind database collects binding data for macromolecules and their ligands, and provides some basic display of protein–ligand complexes [9]. Both NsSNP Loader and PDBbind, however, focus on a single type of biological data.

A unique PyMOL [10] plugin, PyMine was developed to provide a central data-hub that automatically retrieves multiple types of data directly related to drug discovery from various databases and present them in an easily understandable way. It also allows visualization of the protein 3D structure, ligand binding site, and single amino acid variation (SAV) information. Hence, through data integration and visualization, PyMine will provide a broader perspective on the drug discovery project in hand in a limited time frame to help generate new ideas for drug development. Along with other PyMOL plugin tools [11, 12], PyMine could be used as a platform to head start any drug discovery projects.

## Implementation

**Fig. 1:**
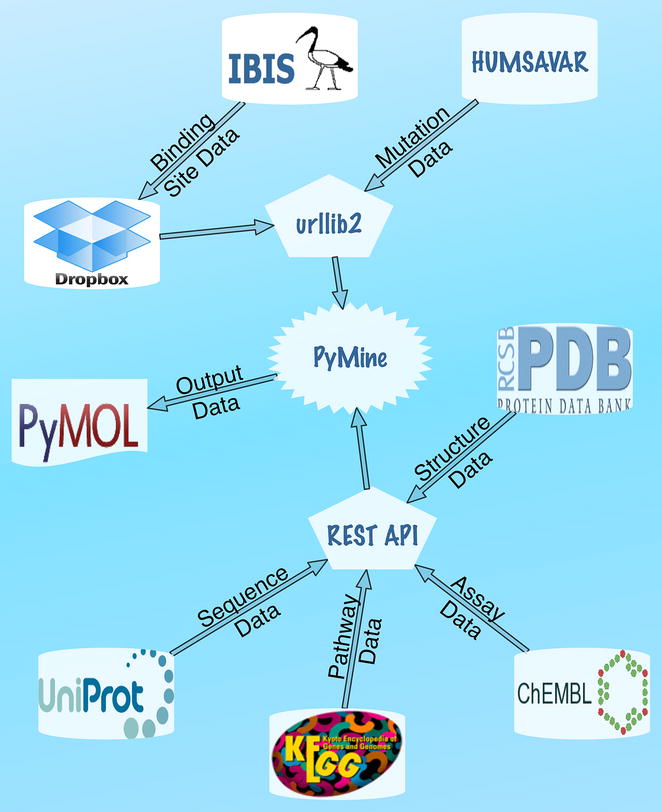
A diagram illustrates database integration in PyMine

We implemented PyMine as a PyMOL plugin that integrates and visualizes chemical and biological data in the 3D visualizer of PyMOL. For data integration, large databases including UniProt [13], PDB [14], CHEMBL [15] and KEGG [16] databases were accessed using REST API (Fig. 1). RESTful web services were selected over traditional SOAP services to minimize the dependence on external libraries. SAV information from the HUMSAVAR database [17] was connected using the urllib2 library. IBIS data [18] were uploaded to our account at Dropbox (www.dropbox.com) and were also connected using the urllib2 library.

For data visualization, PyMine takes advantage of the 3D visualization module of PyMOL that makes it a unique tool to combine visualization of structural data along with presenting relevant biological information.

PyMine was written in python and is compatible with python versions 2.5.6 and above (Python Software Foundation, www.python.org). The GUI was created using Python’s native Tkinter library. For minimal dependence, no external python package/libraries were used. PyMine has been tested on Ubuntu 10.04.5 and Mac OS X 10.7.5 systems.

## Results

We developed PyMine to serve as a central data-hub that automatically retrieves chemical and biological data from multiple databases and presents them in an interactive and uniformed way. To illustrate the function of PyMine, we chose a wild-type protein of the BRAF oncoprotein with known structure (PDB ID: 4E26) as an example. The BRAF oncoprotein is found to be mutated in about half of malignant melanomas and other cancers, and is under intensive studies.

### Data integration

**Fig. 2:**
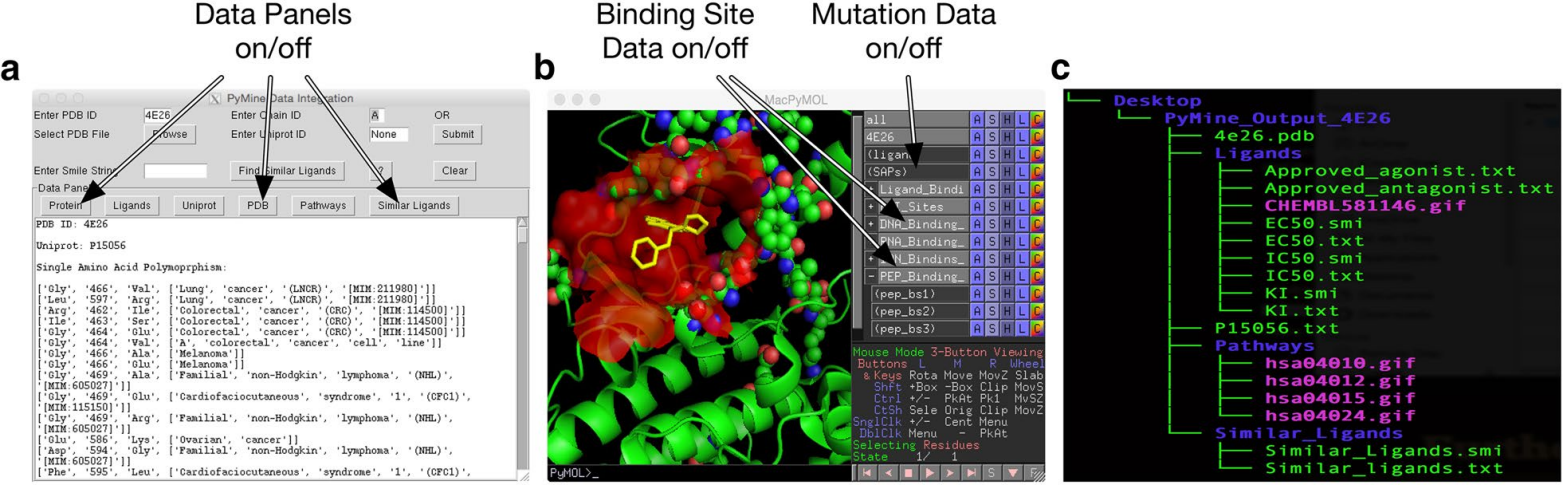
**a** PyMine data integration panel with functional tabs. **b** PyMOL window showing receptor in *green*, ligand in *yellow*, binding site in *red*, and SAVs in *ball* and *stick style*. On *right hand side*, an expandable list of binding sites selections. **c** Output directory and files

In the current version, the only user input required is the PDB ID of the wild-type sequence of the protein of interest. A user is also allowed to input his own protein structure file, along with the UniProt ID. Using the PDB ID of 4E26 as input (Fig. 2a), the PDB structure file of the BRAF protein was fetched from the PDB database [14] and loaded in PyMOL’s graphic visualizer. Since this structure also contains an inhibitor, the available structural information of the inhibitor was also automatically imported. The binding site data of this BRAF protein was obtained from the IBIS database [18]. The binding site information was annotated either from experimental data or based on the binding site information of homologous proteins [18]. The RESTful web services are used to map the PDB ID to the UniProt ID using the pdbtosp.txt file at www.uniport.org/docs/ [13] in order to import the UniProt file of this protein sequence. The UniProt ID is also used to import SAV information from the HUMSAVAR database [17], the approved drug and compound activity data from the CHEMBL database [15], and the pathway information from the KEGG pathway database [16]. All these information can be accessed through the corresponding buttons in the Data Panel (Fig. 2a).

### Data visualization

Through the 3D visualization module of PyMOL, the 3D structure of the BRAF protein was automatically loaded into the graphic window of PyMOL (Fig. 2b). In addition, available SAV and binding site information for the protein was retrieved and also mapped onto the 3D structure of the protein without any extra commands from the user. By toggling among the buttons provided on the right panel, users can selectively display certain information over others for visual study.

### Data output

Along with visualization, PyMine also generated multiple output files containing information such as a list of FDA approved drugs and compounds studied in biochemical assays of the protein of interest and pathway map (Fig. 2c). These files were generated in the standard format and can be used directly for protein–ligand docking studies or network analyses.

## Discussion

Hundreds of biological databases of various types have been generated and many of them are growing rapidly [1, 2]. It is becoming increasingly challenging for an individual biological scientist to mine those databases manually and periodically in order to collect information related to the protein of their interest and to make sense of those data of different types such as protein structure, binding site, SAV, compound and pathway [3]. To help alleviate the problems related to this challenge, we developed the PyMine software that automatically retrieves data from six major and comprehensive, chemical and biological databases so that users can access all the data using a single platform.

Using PyMine as a central data-hub will provide several advantages. First, the users don’t need to access those databases manually and individually since all data will be automatically retrieved. Second, the protein structure and its binding ligand will be automatically loaded into PyMOL’s graphic window with binding site and SAV data mapped onto the structure. Users can visually study them to understand the structural and functional impact of SAV and to develop new ideas about compound modification for structure-based molecular design. Further, Most of the information is outputted into text files in the standard format, which can be directly adopted for further computational studies.

In the current implementation, PyMine focuses on data extraction and visualization. No algorithms are developed and incorporated for further computational analyses such as structure comparison or protein–ligand docking. In addition, information contained in all the databases under consideration except for the PDB database are deposited based on the individual wild-type protein sequence. Therefore, the PDB ID or UniProt ID entered by a user should correspond to the wild-type sequence of the protein. Otherwise, most of the information won’t be retrieved and loaded.

Several efforts have been reported for data visualization and integration [5–9]. Compared to those software packages of similar purpose, PyMine was developed to automatically and exclusively retrieve multiple types of data that are directly related to drug discovery. In addition, PyMine is based on PyMOL, a free and broadly adopted graphic visualization software.

Overall, we reported here a PyMOL plugin that focuses on automatically collecting and integrating multiple types of data from various database sources. With the growth of both database number and size, we envision this plugin will be widely adopted by biological scientists with/without bioinformatics training. Furthermore, PyMine can be combined with other PyMOL plugins such as the Amber and AutoDockVina plugins for molecular dynamics simulations and protein–ligand docking [12], to provide an integrated platform for drug discovery. In the future, we plan to continuously improve PyMine by incorporating additional databases.

## Conclusions

In summary, we proposed an innovative, easy to use PyMOL plugin that automatically retrieves chemical and biological data from six high-quality biological databases and presents them in an easy understandable way. Using a popular graphic software PyMOL, multiple types of data can be accessed easily without going through multiple databases manually. Users can instead focus on understanding those data and use the graphic tools to help develop new hypotheses.

## Availability and requirements

- Project name: PyMine.
- Project home page: https://github.com/zhijunlilab/PyMine.
- Operating system(s): Linux and Mac OS X.
- Programming language: Python.
- License: MIT license.
- Any restriction to use by non-academics: license needed.
